# Methane fluxes from coastal sediments are enhanced by macrofauna

**DOI:** 10.1038/s41598-017-13263-w

**Published:** 2017-10-13

**Authors:** Stefano Bonaglia, Volker Brüchert, Nolwenn Callac, Alessandra Vicenzi, Ernest Chi Fru, Francisco J. A. Nascimento

**Affiliations:** 1 Department of Ecology, Environment and Plant Sciences, Stockholm University, Stockholm, Sweden; 2 Bolin Centre for Climate Research, Stockholm University, Stockholm, Sweden; 3 Department of Geological Sciences, Stockholm University, Stockholm, Sweden; 4 School of Earth and Ocean Sciences, Cardiff University, Cardiff, Wales UK

## Abstract

Methane and nitrous oxide are potent greenhouse gases (GHGs) that contribute to climate change. Coastal sediments are important GHG producers, but the contribution of macrofauna (benthic invertebrates larger than 1 mm) inhabiting them is currently unknown. Through a combination of trace gas, isotope, and molecular analyses, we studied the direct and indirect contribution of two macrofaunal groups, polychaetes and bivalves, to methane and nitrous oxide fluxes from coastal sediments. Our results indicate that macrofauna increases benthic methane efflux by a factor of up to eight, potentially accounting for an estimated 9.5% of total emissions from the Baltic Sea. Polychaetes indirectly enhance methane efflux through bioturbation, while bivalves have a direct effect on methane release. Bivalves host archaeal methanogenic symbionts carrying out preferentially hydrogenotrophic methanogenesis, as suggested by analysis of methane isotopes. Low temperatures (8 °C) also stimulate production of nitrous oxide, which is consumed by benthic denitrifying bacteria before it reaches the water column. We show that macrofauna contributes to GHG production and that the extent is dependent on lineage. Thus, macrofauna may play an important, but overlooked role in regulating GHG production and exchange in coastal sediment ecosystems.

## Introduction

Methane (CH_4_) and nitrous oxide (N_2_O) in the atmosphere constitute a severe threat to Earth’s climate, with up to 28 and 265 times greater warming potential than carbon dioxide (CO_2_), respectively^1^. Human activities such as industrial production, intensive agriculture, and livestock farming have substantially increased the levels of greenhouse gas (GHG) emissions. Recent estimates report a 150% and 20% growth of atmospheric CH_4_ and N_2_O levels, respectively, since 1750, which is unprecedented over the last 800,000 years^1^. Anthropogenic pressures have also strongly altered aquatic ecosystems because agricultural expansion and increased use of synthetic fertilizers have caused extensive nutrient enrichment in near-coastal water^2^. This condition, also known as eutrophication, has been recognized to be the principal driver for the enhanced GHG flux from aquatic environments^3^. According to recent budgets, shallow aquatic systems may contribute ~10% of global N_2_O emissions^4^. There is no clear consensus on the contribution of these environments to the global CH_4_ emission because source magnitude and variability remain highly uncertain^5^. However, up to 30–40% of the methane emissions may be due to methane produced in sediments of aquatic ecosystems^4^.

The largest part of the metazoan biomass in coastal sediments is contributed by macrofauna, i.e., invertebrates with body dimension exceeding 1 mm^6^. Through reworking and bioirrigation, macrofaunal activities profoundly impact biogeochemical processes and microbial diversity^7–9^. In recent years, a debate has arisen whether benthic invertebrates would be effective in counteracting human pressures on aquatic environments^10,11^. Supporters of this paradigm have proposed that bivalve activity may alleviate nutrient loading because of high turnover rates of nutrients by incorporation into shellfish, which are subsequently removed from ecosystem for human consumption^12,13^. Apart from these potential ecological benefits, bivalve farming would be expected to resolve social and economic issues worldwide as mollusk production has accounted for more than 70% of all mariculture since 1970^14^. Critics, however, argue that macrofauna would increase rather than reduce internal nutrient loading because of high ammonium regeneration associated with invertebrate excretion and the stimulation of bacteria carrying out dissimilatory nitrate reduction to ammonium^15–18^. In this discussion, the impact of macrobenthos on GHG release is much less understood.

The role of coastal benthic macrofauna in mediating gas release is still amply debated since the mechanisms regulating production and transport of gases by invertebrates are largely unknown. Recently, however, it was demonstrated that most of these organisms produce N_2_O in their digestive tracts^19^. Thus, bivalves isolated from coastal sediments were shown to be strong emitters of N_2_O^20,21^. However, it is not clear from these studies whether the N_2_O produced by bivalves reaches the water column or is reduced to dinitrogen by denitrifying bacteria living in the sediment. Recent investigations suggest that chironomid larvae significantly stimulate the sedimentary release of N_2_O to the water column^22^, while N_2_O and CH_4_ release does not significantly increase along with tubificid oligochaete abundance^23^. However, a recent study assessing urban wetlands showed that CH_4_ and CO_2_ fluxes correlated with tubificid abundance^24^. Experimental work with manipulated Baltic Sea sediment suggested that bivalves may induce a seven- to ten-fold increase in CH_4_ efflux compared to sediment without macrofauna^16^, but no systematic studies have been conducted to investigate direct CH_4_ production by benthic fauna and to quantify their impact on benthic GHG release.

Here, we report on direct and indirect GHG release from ubiquitous macrofaunal organisms; the bivalve *Limecola balthica* (formerly named *Macoma balthica*); and the polychaete *Marenzelleria arctia*; two of the most common macrofaunal groups inhabiting Baltic Sea sediments. The specific aims of the study were to: (1) test if and by how much macrofauna alters sediment-water fluxes of CH_4_ and N_2_O; (2) quantify direct macrofaunal release of these GHGs; (3) estimate the carbon source of methanogenesis associated with the bivalve microbiome; (4) quantify methanogenic symbionts associated with macrofauna. Our study provides, to our knowledge, the first information to date on direct CH_4_ and N_2_O release by macrofaunal organisms characterized by different functional traits, and their regulation of methanogenic activity in coastal marine sediments.

## Results

### Sediment core experiment for fluxes of methane and nitrous oxide

A sediment core incubation experiment was carried out to quantify macrofauna alteration of sediment-water fluxes of CH_4_ and N_2_O one day (day 1) and ten days (day 10) after macrofauna addition. The sediment used for incubations (0–15 cm layer) had an average carbon content of 5.5% (Volker Brüchert, unpubl. data). Analysis of water samples from the incubation tank revealed that the oxygen concentrations were constant during the ten days of the experiment as they were 348 µM at day 1 and 350 µM at day 10. Ammonium concentrations were always low (<1.2 µM). Concentrations of nitrate changed significantly during the course of the experiment (*P* = 0.003), and were 2.2 ± 0.1 µM at day 1 and increased to 6.2 ± 0.2 µM at day 10. Macrofauna survival after sediment core incubations was 100% for *L. balthica* and 92% for *M. arctia*, which indicates good ecological conditions for these two species during our experiment.

**Figure 1:**
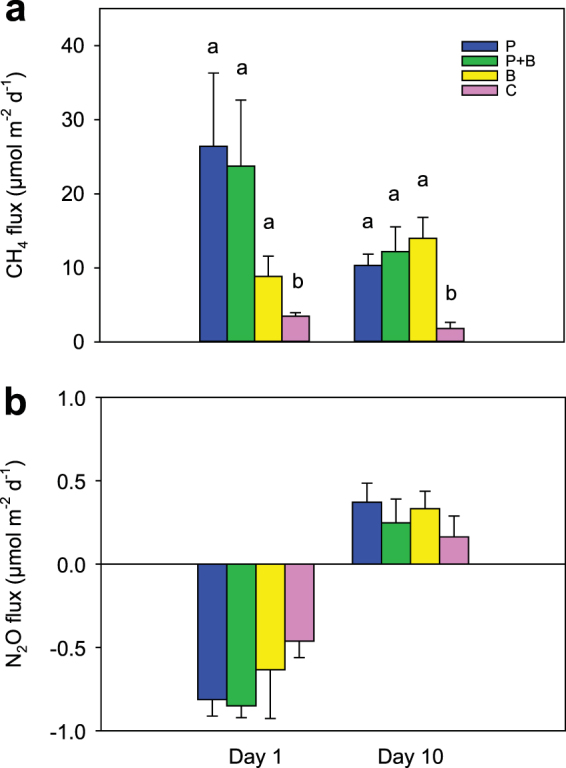
Sediment-water exchange fluxes of methane (**a**) and nitrous oxide (**b**) in the different treatments (P, sediment with polychaetes; P + B, sediment with polychaetes and bivalves; B, sediment with bivalves; C, control sediment) and at different times (Day 1 and 10) determined by intact-core incubations. For methane fluxes, different letters indicate significant differences among treatments, while for nitrous oxide fluxes no significant differences among treatments were found (Table 1). Vertical columns represent average fluxes, while error bars represent s.e.m. (n = 7 per treatment).

Fluxes of CH_4_ were always directed from the sediment to the water column (Fig. 1a), and fluxes in the treatments with macrofauna were significantly higher than those in the control sediments without macrofauna (Table 1). The increase in CH_4_ flux caused by polychaetes was more pronounced at day 1 than at day 10, while bivalves stimulated the CH_4_ flux more at day 10 than at day 1 (Fig. 1a). However, results from the Scheirer-Ray-Hare test showed that the differences in CH_4_ effluxes between day 1 and day 10 were not significant (Table 1). Nitrous oxide fluxes were directed from the water column into the sediment at day 1 (=uptake), while their direction was reversed at day 10 (=efflux) (Fig. 1b). There were no differences in fluxes of N_2_O between treatments, but these fluxes were significantly different between day 1 and day 10 (Table 1).

**Table 1:** Summary of the results of the Scheirer-Ray-Hare test.

Variable	Factor	df	H	*P* value	Significant?
CH_4_ flux	Incubation day	1	0.50	0.481	no
	Treatment	3	23.2	<0.001	yes
	Incubation day X Treatment	3	4.3	0.228	no
N_2_O flux	Incubation day	1	34.6	<0.001	yes
	Treatment	3	1.5	0.686	no
	Incubation day X Treatment	3	1.4	0.698	no

The test was performed to detect the effect of factors *Incubation day* and *Treatment* on gas fluxes from sediment core incubations. Meaning of statistical variables: *df* is degree of freedom; *H* refers to the value of the Scheirer-Ray-Hare test; *P* < 0.05 represents significant differences, while *P* > 0.05 represents no significant differences.

### Methane and nitrous oxide production by macrofauna

Direct quantification of CH_4_ and N_2_O in serum bottles incubated with filtered bottom water and with macrofaunal specimens showed that gas production was detectable and linear over time (i.e., showing no lag phase) (Supplementary Fig. 1). The control treatments of filtered bottom water only (treatments C) did not result in significant increase of CH_4_ and N_2_O concentrations over time (data not shown).

**Figure 2:**
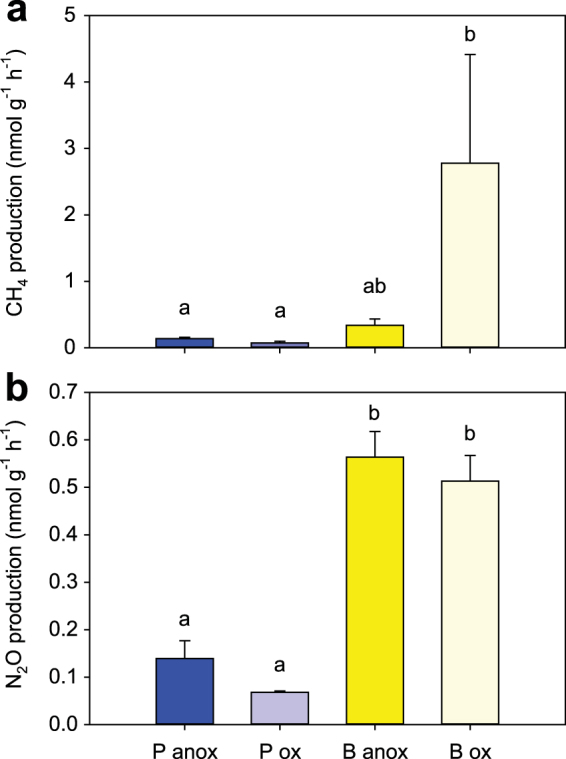
Production of methane (**a**) and nitrous oxide (**b**) from incubation of glass bottles with animals (P, polychaetes; B, bivalves) and either anoxic (anox) or oxic (ox) water. Different letters indicate significant differences among treatments (Table 1). Vertical columns represent average fluxes, while error bars represent s.e.m. (n = 5 per treatment).

CH_4_ production differed significantly among animals (Table 2). The highest values were measured in bivalve incubations under oxic conditions (B ox) (Fig. 2a). These production rates were significantly higher than in both treatments with polychaetes (P anox and P ox), while they were not significantly different from B anox (Table 2). Production of N_2_O was also significantly different among animal treatments (Table 2). Treatments with bivalves produced significantly more N_2_O than treatments with polychaetes (Table 2). Although the animals produced more N_2_O under anoxic than oxic conditions (Fig. 2b), the difference was not statistically significant (Table 2). It is thus clear that *L. balthica* individuals, or the microbes associated with the bivalves, produced more CH_4_ and N_2_O compared to *M. arctia* individuals.

**Table 2:** Summary of the results from the ANOVA tests.

Experiment	Parameter investigated	Test result	*P* value	Significant?	Differences between treatments			
					P	P + B	B	C
Sediment core incubations	CH_4_ flux	*see Table* 1	*see Table* 1	*see Table* 1	a	a	a	b
	N_2_O flux	*see Table* 1	*see Table* 1	*see Table* 1	a	a	a	a
					**P (anox)**	**P (ox)**	**B (anox)**	**B (ox)**
Animal incubations	CH_4_ production	H = 16.6	< 0.001	yes	a	a	ab	b
	N_2_O production	F = 35.3	< 0.001	yes	a	a	b	b
					**B**		**C**	
Isotope analyses of methane	δ^13^C-CH_4_	H = 6.2	0.011	yes	a		b	
					**B (st)**		**B (sat)**	
Starvation experiment	*mcrA* gene quantity	F = 1.5	0.235	no	a		a	

One-way parametric (F values) and non-parametric Kruskal-Wallis analysis of variance (H values) were performed to test differences among treatments. The pairwise comparison was performed by means of Tukey test. Different letters represent significant differences (*P* < 0.05), while the same letter represents no significant differences (*P* > 0.05) between treatments. Treatment codes: P refers to treatments involving polychaetes, P + B, involving both polychaetes and bivalves; B, involving bivalves**;** C refers to control treatments; anox = anoxic; ox = oxic; st = starved; sat = satiated. See the Methods for more details about the different treatments.

### Methane carbon isotope composition and *mcrA* genes in *L. balthica*

The δ^13^C-CH_4_ in the control bottles (average ± standard error) representing the composition of bottom water, was −50.1 ± 2.1‰, while the δ^13^C-CH_4_ in the bottles containing CH_4_ associated with bivalve activity was −55.1 ± 0.6‰. There was a significant decrease in the δ^13^C-CH_4_ signal from the bottom water without bivalves to water with methanogenesis associated with bivalves (Table 2). Animals kept in anoxic conditions lead to δ^13^C signatures that were more negative (−56.3 ± 0.2‰) than those associated with animals incubated in oxic conditions (−53.4 ± 0.4‰).

Abundances of *mcrA* genes were 1.4 × 10^10^ ± 3.5 × 10^9^ g^−1^ wet weight (average ± standard error; n = 10) in the body of starved bivalves and 8.3 × 10^9^ ± 2.3 × 10^9^ g^−1^ wet weight (average ± standard error; n = 10) in the body of satiated bivalves (Supplementary Fig. 2). The results of ANOVA tests show that there was no statistical difference in *mcrA* gene abundances between starved and satiated bivalves (Table 2).

## Discussion

This study shows that the efflux of methane from coastal marine sediments is enhanced after colonization by macrofauna. Polychaetes of the genus *Marenzelleria* can colonize the deep sediment layers down to 7–15 cm depth^15,25^, which coincides with the zone of sedimentary methane production in these low saline coastal sediments^26,27^. Our results indicate that polychaetes mobilize pore-water methane and increase the methane efflux from the sediment to the water column right after their colonization by a factor of eight compared to bare sediments. This pore-water flushing is also seen when polychaetes recolonize sediments together with bivalves because the methane efflux in these treatments was seven times higher than in sediments without macrofauna. These findings substantiate those from a previous study reporting a positive correlation between benthic methane flux and polychaete biomass in the sediment^15^ and suggest that the flush-out effect described for porewater nutrients^28^ is also effective for methane.

The bivalve *L*. *balthica* normally is active in the upper 2–5 cm of sediment in the Baltic Sea^16,29^, where pore-water methane concentrations are usually low^26,27^. Thus, it is likely that bivalves do not induce the same immediate effect on methane efflux after colonization as polychaetes and that the increase in the methane flux (up to a factor of eight compared to bare sediments) may be due to methanogenesis directly associated with the bivalve^16^. This hypothesis is further corroborated by the results from the *in vitro* animal incubations that show considerable production of methane by the bivalve *L. balthica*, and to a lesser extent by the polychaete *M*. *arctia* (Fig. 2A). These results, together with the high abundances of *mcrA* genes quantified in both starved and satiated bivalves, indicate that the bivalve body, i.e., the anoxic intestine, is colonized by active methanogens (Supplementary Fig. 2). Thus, both macrofaunal groups contribute to methane release, but the extent and mechanisms behind the stimulation are dependent on the different functional traits.

**Figure 3:**
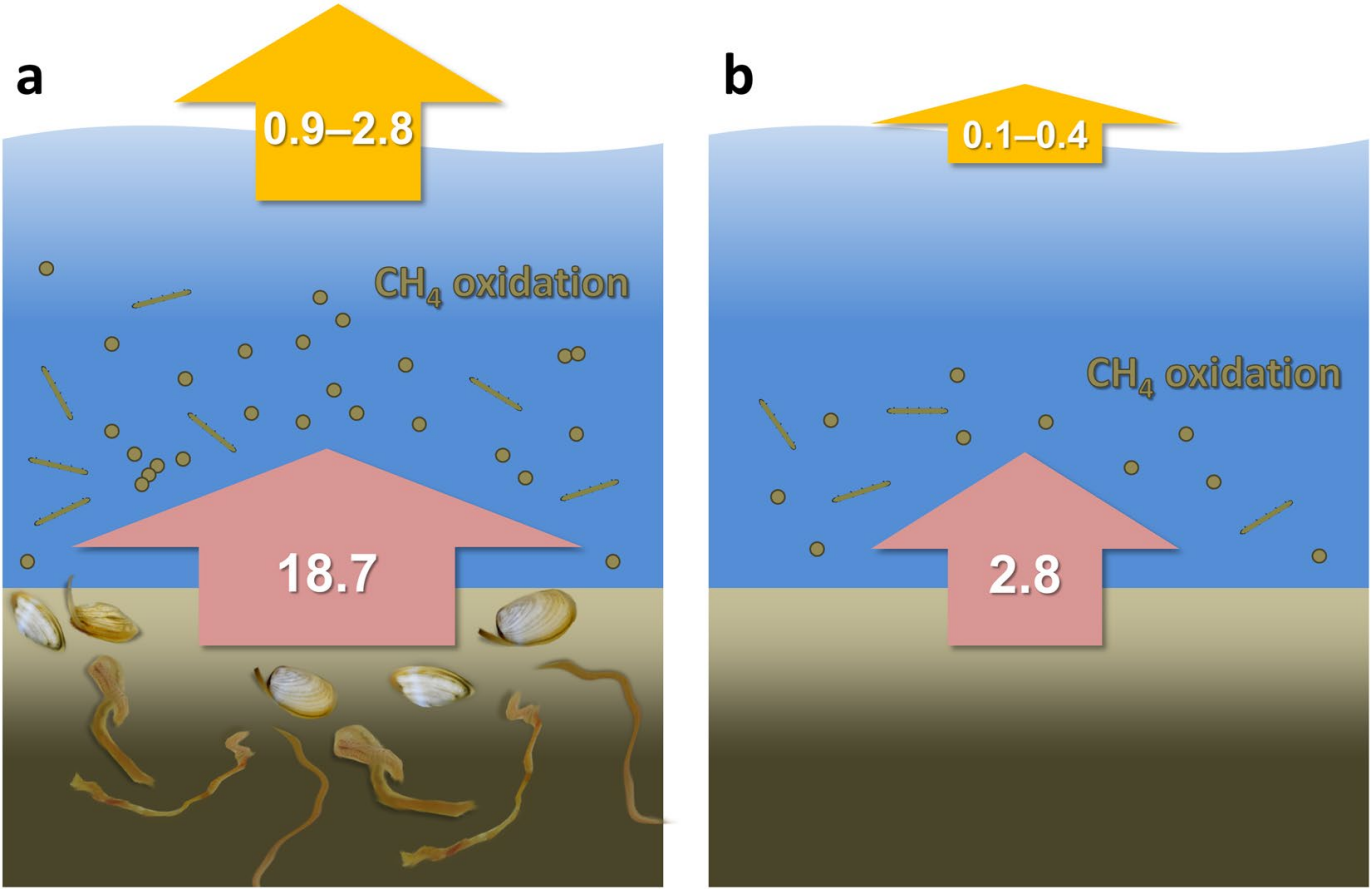
The influence of macrofauna on benthic methane flux and emission to the atmosphere in the Baltic Sea. Pink arrows depict the benthic fluxes of methane in the current situation with macrofauna (**a**), and in the scenario of sediments devoid of macrofauna (**b**). Orange arrows represent estimated emissions of methane to the atmosphere. Benthic fluxes and emissions are expressed in Gg C year^−1^. For more information see the Discussion.

Our study presents evidence for direct macrofauna GHG release from *in vitro* incubations at low *in situ* temperatures, contrary to previous studies in which animals were incubated at room temperature^19,20^. Our investigations also confirm that coastal marine invertebrates release nitrous oxide, which may be produced by microorganisms that are assimilated with the ingested food^19,20^. Production rates of nitrous oxide by *L. balthica* were about half those reported in a previous study investigating the same organism^20^, and this difference may due to the lower temperature selected for our experiment (8 °C) compared to the former assessment (21 °C).

Recent studies revealed that nitrous oxide production is associated with the activity of denitrifying bacteria present in the anoxic gut of aquatic invertebrates, and depends on the nitrate concentration in the gut^22,30,31^. Nitrate concentrations in the gut of nitrous oxide-producing macrofauna typically range from a few to several hundred µM^30,32^. We did not quantify the gut nitrate concentration in this study, but based on the analysis of the incubation water, nitrate was always present at concentrations of 8–9 µM, which was sufficient to sustain denitrification in the anoxic gut of the invertebrates. Nitrification can also lead to nitrous oxide production, but this process requires oxygen and has so far only been described in shell biofilms^33,34^. Hence, we cannot exclude that part of the nitrous oxide produced by bivalves was due to nitrification. Body weight has been suggested to be the main factor correlating with nitrous oxide production, as larger animals have larger guts and ingest more microbes than smaller organisms^20^. Since the invertebrates in our study had similar body weights this factor seems improbable, and nitrous oxide production was more likely mediated by the invertebrate feeding mode. *L. balthica* feeds mainly on deposited phytodetritus, suspended particles, and microorganisms present in the top centimeters of the sediment, where denitrifiers are abundant. *M. arctia* is a strict deposit feeder and lives buried in deeper, often sulfidic sediment layers^15^, which suggests that its diet may be poor in denitrifiers.

The results from the core experiments show that the initial benthic uptake of nitrous oxide reversed to a benthic efflux after ten days (Fig. 1b). High nitrate concentrations lead to high and almost exclusive nitrous oxide yields in nature^35,36^. The nitrous oxide uptake after one day might thus be due to the low nitrate concentrations (2.2 µM) in the overlying water. As macrofauna produced nitrous oxide in our investigations, it is likely that it was consumed by bacterial denitrification before reaching the overlying water, as denitrification (but not nitrification) can be a sink for nitrous oxide^37^. Higher nitrate concentrations (6.2 µM) in the overlying water after the ten-day period were associated with a nitrous oxide efflux in all treatments. We interpret the higher concentration to be due to an increase in nitrification activity associated with a larger surface area by newly created burrow walls^38^. Both the higher concentrations of nitrate and the increase in nitrification activity may have contributed to the net benthic release of nitrous oxide with time. Although this appears to be the most plausible explanation for our results with the available data, the reversal of benthic nitrous oxide flux with acclimatization of the animals to experimental conditions should be addressed in depth in future investigations as this phenomenon comes along with important environmental implications.

Biogenic methane is produced by archaea under anoxic conditions primarily by carbon dioxide reduction coupled to hydrogen oxidation (hydrogenotrophic methanogenesis) resulting in strongly ^13^C-depleted methane (more negative δ^13^C) or by acetate fermentation (acetotrophic methanogenesis) resulting in relatively less ^13^C-depleted methane (less negative δ^13^C)^39^. In lacustrine sediments, acetotrophy is more important than hydrogenotrophy^39^, while in the Baltic hydrogenotrophic methanogenesis rates can be up to one order of magnitude higher than those of acetate methanogenesis^40^. In the case of ruminants and digestive tracts of humans and insects hydrogenotrophy also prevails (cf. Fig. 3 in ref.^41^). The isotopic signature of the methane produced by bivalves was significantly more depleted in ^13^C than its surrounding water, which may imply a stronger contribution of hydrogenotrophic methanogenesis in marine invertebrates than in the surrounding sediment. We cannot exclude that methane oxidation co-occurred during incubation, which would make the residual methane enriched in ^13^C^42^. In incubations done in anoxic, helium-purged waters, all the residual CH_4_ was produced by the invertebrates. Values from these anoxic incubations were slightly more negative (−56.3‰) than the others that were oxic from the start (−53.4‰). The isotopic signal from oxic incubations was almost exclusively (>90%) due to CH_4_ produced by the invertebrates, indicating that the difference (2.9‰) could be attributed to methane oxidation activity.

Analysis of the *mcrA* gene has successfully been applied to monitor methanogens in soil invertebrate guts^43,44^. Detection of *mcrA* genes in starved bivalves suggests that the methane was not produced by ingested microbes. However, locating the methanogens would only have been possible with, for example, FISH^45^. Regardless, our results show similar abundances of *mcrA* genes between starved and satiated bivalves, indicating that methanogens were not associated with ingested food, but were rather symbionts colonizing the inner, anoxic digestive system (i.e., gut) as for other invertebrates^43,44,46^. The high variations in abundances of *mcrA* genes between different specimens (Supplementary Fig. 2) may explain the high variation in methane produced by bivalves in oxic conditions (Supplementary Fig. 1). Thus, methane producers can be found in 30–50% of human intestines^47^. Because of this trait, about half of humanity has the capacity to produce methane. Based on the high gene and rate variations, we cannot exclude that also invertebrate guts may either have or not have the capacity to produce conspicuous amounts of methane.

A symbiosis between methane-cycling microorganisms and bivalves was previously reported^48^, but the microbes were dependent on, rather than producing, methane. In our study, the symbiosis might be based on hydrogen gas produced in the intestinal tract by fermentation, also in light of the fact that hydrogenotrophy prevailed when animals were present. As in the case of rumen symbiosis, the animals might benefit from the microbiota converting refractory polymers into more labile compounds, which can be digested and absorbed by the animal^49^. Methanogens are strict anaerobes but the average methane production by bivalves was higher in oxic than in anoxic conditions (Fig. 2a), although this difference was not significant because of the high variation in methane production in oxic conditions (Supplementary Fig. 1). Anoxia is expected to inhibit bivalve activity because the proliferation of fast-growing sulfate-reducing bacteria (SRB) in the incubation medium would damage the animal tissues^50^. SRB outcompete methanogens for the mutual substrate hydrogen both in intestines^51^ and in sediments^52^. These observations suggest that the symbiosis was less beneficial under strictly anoxic conditions when bivalve performance was likely reduced and the archaeal symbionts were outcompeted by SRB^50^.

Sediments underlying oxic water in the Baltic Sea are dominated by a macrofaunal community characterized by *Marenzelleria* spp. and *Limecola balthica* ^53,54^. By scaling up our benthic methane flux from sediments inhabited by the *Marenzelleria*/*Limecola* community (12.2 µmol C m^−2^ d^−1^) to the area of the Baltic Sea with oxygenated bottom water (349,133 km^2^)^55^, the extrapolation results in a total benthic flux of 18.7 Gg C year^−1^ from sediments inhabited by macrofauna (Fig. 3). This study did not quantify rates of methane consumption in the water column, but literature suggests that methane concentrations are reduced, due to efficient methane oxidation, by 85–95% in the Baltic sea coastal area (20–25 m)^56,57^. Considering that the average depth of the oxic Baltic Sea is well within this depth range of 20–25 m, we conclude that macrofauna-inhabited sediment may be a source of atmospheric methane ranging between 0.9 and 2.8 Gg C year^−1^. If the Baltic sediments were devoid of macrofauna these would only contribute to emissions ranging between 0.1 and 0.4 Gg C year^−1^ (Fig. 3). Thus, with the caveats that field-based methane oxidation experiments are needed and that macrofaunal communities are more complex than we assumed here, our results indicate that this contribution should be taken into account in biogeochemical models. In the case of the Baltic Sea, the emission caused by macrofauna would represent an average of 9.5% of the overall methane emission, which was estimated to be 0.02 Tg C year^−1^ ^58^.

Our experimental data together with these extrapolations provide evidence that sediment macrofauna contributes significantly to GHG effluxes from coastal marine sediments. We propose that the macrofaunal GHG contribution may be of particular importance in shallow-water environments, where the gas is emitted to the atmosphere from oversaturated waters e.g.^27,58,59^. More systematic studies should be carried out to investigate the impacts of different invertebrate traits and activities on biogeochemical processes. These experiments should analyze the production of climate-important metabolic products, such as methane, nitrous oxide, carbon dioxide by invertebrates, but also include overlooked climate stressors such as nitric oxide, when it comes to deciding whether to use, for example, bivalve farming as a nutrient reduction measure in the marine environment.

## Methods

### Sampling

Sampling was performed at two coastal sites in the Baltic Sea: sediment and bottom water were collected in Tvären Bay (50 m depth; 58°46′N 17°25′E), while macrofaunal specimens of *Limecola balthica* and *Marenzelleria arctia* were collected in Uttervik (28 m depth; 58°50′N 17°31′E). Sediment was collected with a boxcorer, bottom water (salinity 6.8; temperature 8.0 °C) with a Niskin bottle and macrofauna with a benthic sledge. Tvären sediments are naturally poor in macrofauna because the basin undergoes seasonal hypoxia in late summer and autumn. However, in spring, the bottom water was fully saturated with oxygen and the sediment was oxidized down to 3–4 cm depth. Immediately after collection, samples were transported to the Stockholm University Marine Research Centre at Askö (Stockholm archipelago), where they were placed in a climate-controlled room at *in situ* temperature. All experiments were conducted at 8 °C in the laboratories of Stockholm University.

### Bioturbation effect on methane and nitrous oxide fluxes

Two incubations of sediment cores with and without macrofauna specimens were carried out to test the effect of macrofauna bioturbation on gas fluxes one day after animal addition and after ten days of acclimation. Sediment cores (n = 28; 4.6 cm inner diameter and 30 cm length) were placed in a sediment core incubator filled with bottom water. Stirring mechanisms and water pumps were added to keep the water oxygenated. Macrofauna specimens were added to the sediment cores, which were intact and naturally devoid of macrofauna. These experimental units were assigned to four different treatments (n = 7 per treatment): (1) microcosms with addition of six polychaetes (*M. arctia*) in each sediment core (P); (2) microcosms with addition of three polychaetes and three bivalves (*L. balthica*) (P + B); (3) microcosms with addition of six bivalves (B); (4) microcosms consisting of intact, non-manipulated sediment cores (C). The manipulated macrofaunal abundance was 3612 ind. m^−2^, which lies in the range of the abundances recently reported for the Baltic Sea^53,54,60^. The average weight of *M. arctia* and *L. balthica* specimens were not significantly different (p > 0.05; ANOVA) and were 34.7 ± 6.9 and 33.3 ± 8.7 mg wet weight (WW) ind.^−1^ corresponding to 1.7 ± 0.3 and 2.4 ± 0.4 mg dry weight (DW) ind.^−1^, respectively. This resulted in biomasses of *M. arctia* (21 g WW m^−2^) and *L. balthica* (20 g WW m^−2^) that were similar to those reported for the oxic Baltic Sea basins^53,54^.

After one-day of acclimation (day 1), fluxes of methane (CH_4_) and nitrous oxide (N_2_O) between the sediment and the water column were experimentally determined following the procedure described in Bonaglia *et al*.^16^. Briefly, each microcosm was capped with rubber stoppers, while avoiding trapping gas bubbles, and its overlying water was stirred with magnetic stirrers. Water samples for CH_4_ and N_2_O concentrations were taken at the beginning and the end of the incubation, transferred to 12 mL Exetainer vials (Labco Scientific) and biological activity was stopped by adding 100 μL ZnCl_2_ (7 M). Oxygen (O_2_) concentrations were monitored before and after the incubation in each microcosm using a calibrated mini sensor (OX-500, Unisense, Denmark). The incubations always consumed ≤27% (on average 18%) of the initial O_2_ saturation value. The incubation was terminated after 8 h when the microcosms were left uncapped while stirring to return to O_2_ saturation. After ten days of acclimation (day 10), a second incubation experiment was carried out observing the same conditions as day 1, but the incubation time was increased to 10 h. Ammonium (NH_4_^+^) and nitrate (NO_3_^−^) samples (n = 5) were collected from the water tank at day 1 and at day 10 and immediately filtered using 0.2 μm polyethersulfone (PES) filters.

Concentrations of CH_4_ and N_2_O in the water samples were analyzed by headspace analysis on a gas chromatograph (SRI 8610 C) equipped with a flame ionization detector for CH_4_ and an electron capture detector for N_2_O using dinitrogen (N_2_) as carrier gas^59^. Precision was ±1 nM and ±0.2 nM for CH_4_ and N_2_O concentrations, respectively. Net fluxes across the sediment–water interface were calculated from the difference in concentrations in the water column through the incubation period^16^. Concentrations of NH_4_^+^ and NO_3_^−^ were determined colorimetrically on a segmented flow nutrient analyzer system (OI Analytical, Flow Solution IV).

### Direct gas production associated with macrofauna

Glass bottles (n = 30; 50 mL volume) with oxic and anoxic filtered *in situ* water were incubated with and without the addition of animals to check if macrofauna symbionts were producing CH_4_ and N_2_O. Specimens of *L. balthica* and of *M. arctia* were carefully washed three times with 0.2 µm-filtered bottom water. The incubation bottles were assigned to six treatments (n = 5 replicates each treatment): (1) vials with five bivalves in oxic water (B ox); (2) vials with five polychaetes in oxic water (P ox); (3) control vials with oxic water (C ox); (4) vials with five bivalves in anoxic water (B anox); (5) vials with five polychaetes in anoxic water (P anox); (6) control vials with anoxic water (C anox).

All bottles were prepared with 5 mL glass beads (1 mm Ø) that served as digging substratum for infauna, and received either 15 mL of 0.2 µm-filtered oxic (treatments B, P and C ox) or 15 mL of filtered anoxic water (treatments B, P and C anox), which was prepared by bubbling bottom water with a mixture of He (99.9%) and CO_2_ (0.1%) for 20 min and did not alter the *in situ* pH and alkalinity significantly. Glass bead sizes <1 mm were avoided because they may be ingested and retained by benthic macrofauna^61^. After the bottles were capped with butyl septa, water in the anoxic treatments was additionally bubbled for 10 min in order to avoid any potential O_2_ contamination^59^.

The bottles were incubated in a temperature-controlled water bath at 8.0 ± 0.1 °C. Headspace samples were retrieved four times from each bottle during an incubation period of 16 h. Samples were directly injected for CH_4_ and N_2_O concentrations following the procedure described above. Concentrations of CH_4_ and N_2_O in the bottle headspace were used to calculate the animal production over time and were standardized per g WW. Average animal biomasses are reported in the section above.

### Methane isotopes to discriminate CH_4_ production pathways in *L. balthica*

Seven random bottles from the previous experiment that contained bivalves were further processed for stable carbon isotope composition of CH_4_ to differentiate between different pathways of CH_4_ production (i.e., the carbon source) associated with the bivalve microbiome. Seven bottles filled with *in situ* bottom water were considered as controls. Because of low CH_4_ concentrations in bottles with polychaetes and limited financial resources, these were not analyzed for their carbon isotope composition. The analysis was made using continuous-flow gas chromatograph isotope-ratio mass spectrometry at the Stable Isotope Laboratory at Stockholm University, following the protocol described in Wik^62^. Briefly, a Trace GC Ultra gas chromatograph was connected via the Conflo IV to a DeltaV plus mass spectrometer (Thermo Scientific). The Trace GC was equipped with a Split/Splitless injector, a 25 m PoraPlot Q capillary column (Varian, Inc.), and a combustion oven that oxidizes CH_4_ to CO_2_.

Injections were made manually using a 100 µL glass gas-tight syringe (Hamilton, USA) and a sample split on the injector. The injection volume varied from ∼10–80 µL depending on sample CH_4_ concentration. Injections of 5 µL standard (100% CH_4_) were made before the first and after the last sample. The carbon isotopic signature of CH_4_ was calculated as standard delta (δ)-notations relative to Vienna Pee Dee Belemnite (VPDB) using the following equation:

$${\delta }^{13}{\rm{C}}\,=(\frac{{\rm{R}}}{{{\rm{R}}}_{{\rm{VPDB}}}}-1)\times 1000{\rm{\textperthousand }}$$

where R is the ^13^C/^12^C ratio in the samples and R_VPDB_ is the ratio in the VPDB standards. The δ values are expressed in per mil (‰). The analytical precision was 0.3‰.

### Detection of methanogens in *L. balthica*

To detect and quantify methanogenic symbionts inside macrofaunal guts or other tissues, entire specimens were processed for quantitative Polymerase Chain Reaction (qPCR) analysis. Since bivalves produced more CH_4_ than polychaetes, and because of limited financial resources, this analysis was carried out for bivalves only. Briefly, bivalve specimens (n=20) were carefully washed five times in 0.2 µm filtered bottom sea-water. Ten specimens were directly frozen at −80 °C, while ten other specimens were placed in clean water, i.e., in a 0.2 µm-filtered bottom-water bath in a climate-controlled room at *in situ* temperature (8 °C) for 15 h to clean their gut. Water was replaced twice during the gut-cleaning process to avoid feces. After cleaning and three rinsing steps in 0.2 µm filtered water, the animals were frozen at −80 °C.

In the laboratory, the soft parts of the animals were carefully removed from the shells and washed in sterile seawater. The DNA was extracted using the DNeasy Blood and Tissues Kit (QIAGEN), following the manufacturer’s instructions. The detection and quantification of the key gene for methanogenesis, *mcrA* (encoding the alpha subunit of the methyl-coenzyme M-reductase), was determined using specific primers ME3MF (ATGTCNGGTGHGTMGGSTTYAC) and ME2r′ (TCATBGCRTAGTTDGGRTAGT)^63,64^ in 35 cycles at an annealing temperature of 60 °C. Q-PCR conditions were: 500 nM of each primer, 5 µL of DNA template, 12.5 µL of SsoAdvanced^TM^ Universal SYBR^®^ Green Supermix (Bio-Rad) and, following the manufacturer’s recommendations, nuclease-free, sterile deionized water was added to a final volume of 25 µL.

The standard curve was calibrated in ten-fold dilutions ranging from 10^0^ to 10^−5^ using DNA from *Methanoculleus marisnigrii* (DSMZ 1498). All reactions were realized in 96 well Q-PCR plates using CFX96 Touch™ Real-Time PCR Detection System Instrument (C1000 Touch™ Thermal, Cycler, Bio-Rad) and its software. Q-PCR quantifications of *mcrA* genes in sample extracts and in standard series were performed in triplicate alongside with negative controls to rule out laboratory contamination. The total gene copy numbers per gram of animal were calculated from the triplicate sample averages as previously described^65^, and by estimating one copy of the *mcrA* gene per genome. The qPCR amplification efficiency was 96.3% and the slope was −3.41.

### Data analysis

Statistical tests were performed in order to detect differences in the investigated parameters among treatments. Homogeneity of variance of the dataset was checked using Cochran’s test. When ANOVA assumptions were met, one-way analysis of variance tests were performed. When the variance was found to be heterogeneous, non-parametric tests were used (Kruskal-Wallis test). Pairwise post hoc comparisons among treatments were performed by Tukey HSD test. A 2-way non-parametric ANOVA (Scheirer-Ray-Hare test) with incubation day and treatment as factors was performed for testing differences in gas fluxes from sediment core incubations. The Mann-Whitney Rank Sum test was performed to assess differences in nitrate concentrations between day 1 and day 10. Statistical analyses were performed with SigmaPlot 13.0 (Systat Software, CA, USA). If not stated otherwise in the text, measurements are reported as average ± standard error (s.e.m.).

### Data availability

Additional supporting data to the article can be found in the supplementary material. All raw data are available upon request by email to the corresponding author.

## Electronic supplementary material

Supplementary Material
