# Supplementary material for: Methane fluxes from coastal sediments are enhanced by macrofauna
Source: Sci Rep. 2017 Oct 13;7:13145. doi: 10.1038/s41598-017-13263-w (PMC5640653; doi:10.1038/s41598-017-13263-w)
Supplement: Supplementary file 1 — Supplementary Material [file 41598_2017_13263_MOESM1_ESM.doc]

# Methane fluxes from coastal sediments are enhanced by macrofauna

Stefano Bonaglia^1,2*^, Volker Brüchert^2,3^, Nolwenn Callac^3^, Alessandra Vicenzi^3^, Ernest Chi Fru^3,4^, Francisco J. A. Nascimento^1^

^1^ Department of Ecology, Environment and Plant Sciences, Stockholm University, Stockholm, Sweden

^2^ Bolin Centre for Climate Research, Stockholm University, Stockholm, Sweden

^3^ Department of Geological Sciences, Stockholm University, Stockholm, Sweden

^4^ School of Earth and Ocean Sciences, Cardiff University, Cardiff, Wales, UK

^*^ Corresponding author with email stefano.bonaglia@su.se

**--- Supplementary material ---**

## Supplementary Figure 1

##
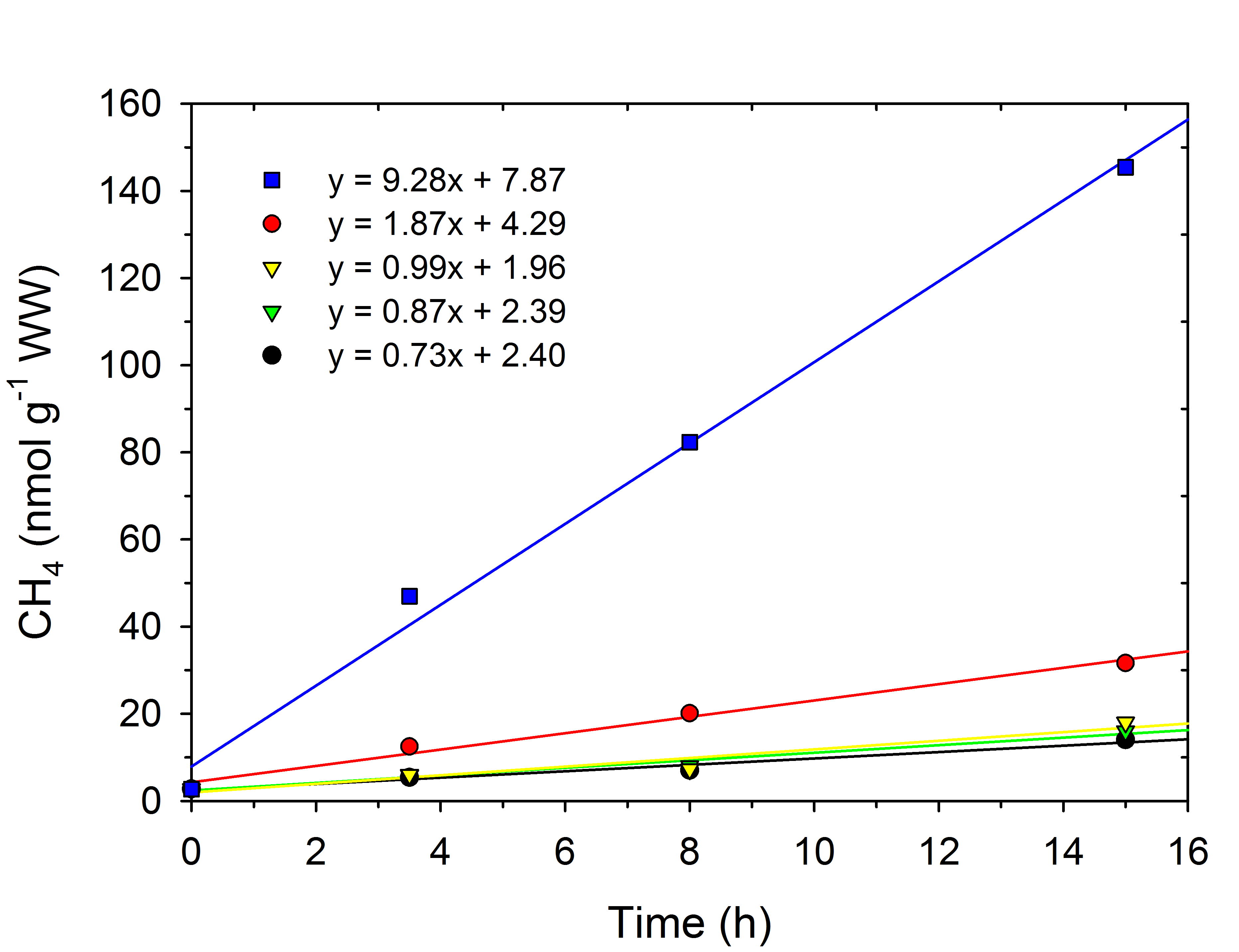


**Supplementary Figure 1:** Examples of methane (CH_4_) production curves (n=5) associated with bivalve specimens (*L. balthica*) incubated in oxic conditions (treatment B ox). Each of the production curves is represented by a color and is accompanied by the relative linear regression analysis results.

## Supplementary Figure 2


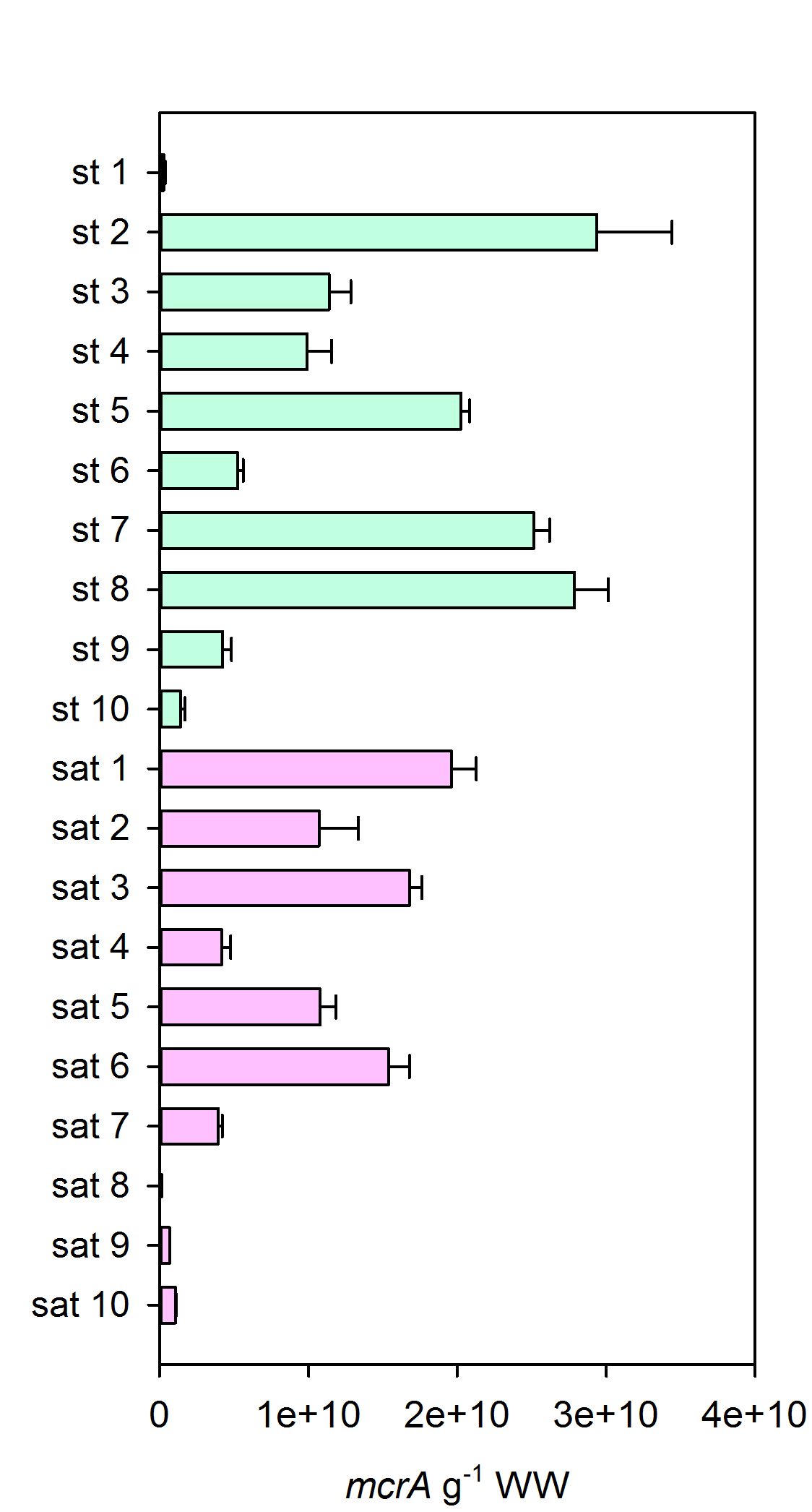


**Supplementary Figure 2:** Abundances of *mcrA* genes per g animal wet weight in each of the digested starved (st) or satiated (sat) specimens of the bivalve *L. balthica*. Horizontal columns represent average gene abundances, while error bars represent standard errors (n=3 per specimen).
